# PM_2.5_-Bound Organophosphate Flame Retardants in Hong Kong: Occurrence, Origins, and Source-Specific Health Risks

**DOI:** 10.1021/acs.est.3c04626

**Published:** 2023-09-11

**Authors:** Xuemei Wang, Chin Wai Leung, Zongwei Cai, Di Hu

**Affiliations:** † Department of Chemistry, Hong Kong Baptist University, Kowloon Tong, Kowloon, Hong Kong 999077, P. R. China; ‡ State Key Laboratory of Environmental and Biological Analysis, Hong Kong Baptist University, Kowloon Tong, Kowloon, Hong Kong 999077, P. R. China; § HKBU Institute of Research and Continuing Education, Shenzhen Virtual University Park, Shenzhen 518057, P. R. China

**Keywords:** organophosphate flame retardants (OPFRs), PM_2.5_, source apportionment, source-specific health risk assessment, plastic processing and waste disposal

## Abstract

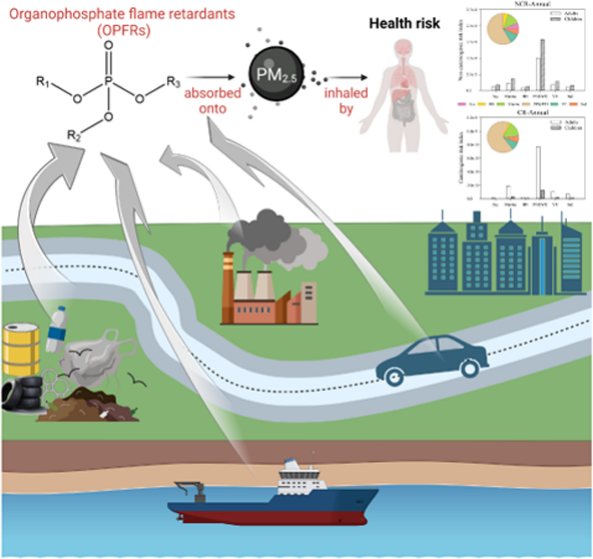

Organophosphate flame retardants (OPFRs) are emerging organic pollutants in PM_2.5_, which have caused significant public health concerns in recent years, given their potential carcinogenic and neurotoxic effects. However, studies on the sources, occurrence, and health risk assessment of PM_2.5_-bound OPFRs in Hong Kong are lacking. To address this knowledge gap, we characterized 13 OPFRs in one-year PM_2.5_ samples using gas chromatography–atmospheric pressure chemical ionization tandem mass spectrometry. Our findings showed that OPFRs were present at a median concentration of 4978 pg m^–3^ (ranging from 1924 to 8481 pg m^–3^), with chlorinated OPFRs dominating and accounting for 82.7% of the total OPFRs. Using characteristic source markers and positive matrix factorization, we identified one secondary formation and five primary sources of OPFRs. Over 94.0% of PM_2.5_-bound OPFRs in Hong Kong were primarily emitted, with plastic processing and waste disposal being the leading source (61.0%), followed by marine vessels (14.1%). The contributions of these two sources to OPFRs were more pronounced on days influenced by local pollution emissions (91.9%) than on days affected by regional pollution (44.2%). Our assessment of health risks associated with human exposure to PM_2.5_-bound OPFRs indicated a low-risk level. However, further source-specific health risk assessment revealed relatively high noncarcinogenic and carcinogenic risks from chlorinated OPFRs emitted from plastic processing and waste disposal, suggesting a need for more stringent emission control of OPFRs from these sources in Hong Kong.

## Abstract 2

Chlorinated OPFRs emitted from plastic processing and waste disposal pose potential noncarcinogenic and carcinogenic risks.

## 1 Introduction

The toxicity of PM_2.5_ is mainly associated with its chemical components. Therefore, it is crucial to study the characterization, source apportionment, and health risk of PM_2.5_-bound toxic substances, such as organophosphate flame retardants (OPFRs), from both environmental and public health perspectives. OPFRs have been widely used as alternatives to polybrominated diphenyl ethers (PBDEs) in recent years. They are semivolatile and can partition onto ambient particles after being released into the atmosphere. Several investigations have reported the toxic effects of OPFRs on organisms upon exposure. For example, triphenyl phosphate (TPHP) has been found to impair heart development in zebrafish by disturbing related gene expression regulators.^1^ Several chlorinated OPFRs, namely, tris(1-chloro-2-propyl) phosphate (TCPP), tris(2-chloroethyl) phosphate (TCEP), and tris(1,3-dichloro-2-propyl) phosphate (TDCPP), were found to be carcinogenic and neurotoxic.^1−3^ Consequently, the European Union (EU) and some states in the United States (U.S.) have promulgated bans on the use of TCEP and TDCPP.^4^

Inhalation is the primary route of human exposure to OPFRs. Several studies have reported the ubiquitous occurrence of chloro-, alkyl-, and aryl-substituted OPFRs (Cl-OPFRs, alkyl-OPFRs, and aryl-OPFRs) in PM_2.5_ samples worldwide, including in Stockholm, Sweden,^5^ Toronto, Canada,^6^ an e-waste site in Pakistan,^7^ and a few cities in China.^7−10^ However, previous studies using gas chromatography electron ionization mass spectrometry (GC-EI-MS) to analyze OPFRs have limited investigation capability on the whole contamination profiles of OPFRs and their fate and transformation in the atmosphere due to inadequate specificity and sensitivity.^5−9^ Recently, gas chromatography–atmospheric pressure chemical ionization tandem mass spectrometry (GC-APCI-MS/MS) has gained attention for the analysis of trace environmental pollutants such as dioxins,^11^ brominated flame retardants,^12^ and polyaromatic hydrocarbons.^13^ Compared to conventional EI and CI sources, APCI is softer and can produce more intense protonated or deprotonated quasi-molecular ions,^14^ providing improved selectivity and sensitivity through a high signal-to-noise (S/N) ratio and higher detection accuracy. However, its application in OPFR analysis is very limited. A few studies have tried to explore the sources of OPFRs by conducting correlation and principal component analysis of individual OPFRs.^8,15,16^ However, no quantitative source apportionment data of ambient OPFRs have been reported due to the lack of complete contamination profiles of OPFRs and source marker data. Although some studies have evaluated the health risks of PM_2.5_-bound OPFRs,^8,17^ none have identified their high-risk sources. Due to the lack of robust quantitative source apportionment data, no study has ever tried to assess the source-specific contributions to PM_2.5_-bound OPFR-associated health risks.

Hong Kong is a highly urbanized city with established recycling centers for electronic waste due to the closure of major e-waste recycling facilities previously located in nearby cities in Guangdong Province.^18^ A number of studies have explored the ambient levels of OPFRs in the Pearl River Delta (PRD) region,^10,19−22^ with one conducted in Hong Kong using a liquid chromatography–triple quadrupole mass spectrometer (LC-MS/MS).^22^ However, no work has been undertaken on the source profiles and source-specific health risks of PM_2.5_-bound OPFRs in the area. Here, we optimized a sample pretreatment and GC-APCI-MS/MS method to characterize PM_2.5_-bound OPFRs. We successfully apportioned the major sources of OPFRs and quantitatively evaluated the source-specific contributions to both OPFRs and their potential health risks in Hong Kong for the first time, which provides a more health-oriented approach to assist in establishing OPFR control policies in this region.

## 2 Materials and Methods

### 2.1 Chemicals and Regents

Thirteen OPFR authentic standards, i.e., triethyl phosphate (TEP), tripropyl phosphate (TPP), tri-*n*-butyl phosphate (TnBP), tris(2-chloroethyl) phosphate (TCEP), tris(1-chloro-2-propyl) phosphate (TCPP), tris(1,3-dichloro-2-propyl) phosphate (TDCPP), tris(2-butoxyethyl) phosphate (TBOEP), triphenyl phosphate (TPHP), 2-ethylhexyl diphenyl phosphate (EHDPP), tris(2-ethylhexyl) phosphate (TEHP), tri-o-cresyl phosphate (TOCP), tri-m-tolyl phosphate (TMTP), and tri-p-cresyl phosphate (TMPP) were purchased from Dieckmann (Hong Kong). Detailed information on OPFR standards is provided in the Supporting Information (SI, Table S1). Three internal standards, which are tris(2-chloroethyl) phosphate-*d*_12_ (TCEP-*d*_12_), tri-*n*-butyl phosphate-*d*_27_ (TnBP-*d*_27_), and triphenyl phosphate-*d*_15_ (TPHP-*d*_15_) were from Toronto Research Chemicals (Canada). Stock standard solutions were prepared in hexane (ultimate grade) at a concentration of 100 mg L^–1^, stored at 4 °C, and diluted to 1000 ng mL^–1^ before analysis. Standard working solutions were prepared by diluting the stock solution to 0.02, 0.2, 5, 50, 200, and 800 ng mL^–1^ in a solvent (hexane) and stored at 4 °C.

### 2.2 Sample Collection

The 24-h sampling of PM_2.5_ was conducted on the roof (114°15E, 22°13N, about 40 m above the ground) of the Owen Hall Building (West Wing) at Hong Kong Baptist University on the schedule of every 6 days from December 8, 2016, to December 27, 2017. 65 PM_2.5_ samples were collected on the quartz fiber filters (20 cm × 25 cm, Whatman) by a high-volume air sampler (Tisch Environmental Inc., Village of Cleves, OH) at a flow rate of 1.13 m^3^ min^–1^. Before and after sampling, we recorded the time, atmospheric pressure, and temperature. After collection, the sample filter was folded in half with clean tweezers, wrapped with aluminum foil, put into a zip bag, and stored in the refrigerator at 4 °C.

To better understand the influence of air mass on the contamination profiles of OPFRs and their sources, we classified the sampling days into three categories according to the backward trajectory of air masses, including local days with local emissions as the dominant sources, regional days that were mainly affected by air mass from the PRD region, and long-range transport (LRT) days that were affected by air masses from the northeast coast of China.^23^ The detailed classification of sampling days was provided in our previous study.^24^

### 2.3 OPFR Analysis

Elemental carbon (EC), organic carbon (OC), and other source markers were analyzed following our previous methods^23,24^ and are briefly described in Text S1. Concentrations of these chemicals are provided in Table S2.

To analyze OPFRs, 20 cm^2^ of each PM_2.5_ sample filter was cut into small strips, put into a 40 mL amber glass bottle, and spiked with 50 ng of TCEP-*d*_12_, 50 ng of TPHP-D_15_, and 50 ng of TnBP-*d*_27_ as internal standards. Details on the optimization of sample extraction and cleanup are provided in the SI (Text S2). After 45 min of ultrasonic extraction in 30 mL of dichloromethane (DCM, HPLC grade), the extract solution was filtered and reduced to about 1 mL by rotary evaporation. The concentrated extract was further cleaned up by a Florisil column (1 g, 6 cm^3^, Dikma, CA) that was prewashed with 12 mL of ethyl acetate and 12 mL of hexane, and the analytes were eluted with 20 mL of DCM/acetone (1:1, v/v). The eluted solution was then reduced to about 1 mL by rotary evaporation, dried under a gentle stream of nitrogen, and reconstituted in 500 μL of hexane (ultimate grade). All samples were sealed and stored at 4 °C in the dark before analysis.

OPFRs were identified and quantified using an Agilent 7890B gas chromatograph (Agilent Technologies Inc.) equipped with a Xevo TQ-S triple quadrupole mass spectrometer (Waters, U.K.). Details on the parameter settings and analytical conditions of GC-APCI-MS/MS are provided in the SI (Text S3). The retention times of all analytes are listed in Table S3. Multiple reaction monitoring (MRM) mode was applied, and the MRM conditions were optimized for each OPFRs (SI, Text S2). The developed analytical method of OPFRs was validated in terms of linearity, repeatability, limit of detection (LOD), and limit of quantification (LOQ) (SI, Text S3). In brief, the recoveries of OPFRs were in the range of 70–120% (Figure S3). The method LODs and LOQs of OPFRs in PM_2.5_ samples were 0.03–0.65 and 0.10–2.06 pg m^–3^, respectively (Table S4). These proved that the GC-APCI-MS/MS-based analytical method developed here could quantitatively analyze the trace level OPFRs in PM_2.5_ with high accuracy, sensitivity, selectivity, and robustness.

## 3 Results and Discussion

### 3.1 Ambient Concentrations and Temporal Trends of OPFRs

We applied the optimized GC-APCI-MS/MS method to analyze 65 PM_2.5_ samples collected in Hong Kong throughout the sampling year and identified and quantified 13 OPFRs (Table S5). The concentration of total OPFRs (∑_13_OPFRs) was 4962 ± 1380 pg m^–3^ (ranging from 1924 to 8481 pg m^–3^), which was lower than those in other cities in China, such as Guangzhou (∑_11_OPFRs, mean, 15.9 ng m^–3^) and Taiyuan (∑_11_OPFRs, mean, 19.5 ng m^–3^)^8^, yet higher than those in the U.S., such as Cleveland (∑_12_OPFRs, 2100 ± 400 pg m^–3^), Chicago (∑_12_OPFRs, 1500 ± 170 pg m^–3^),^25^ and Houston (∑_12_OPFRs, ranging from 160 to 2400 pg m^–3^).^26^

The two Cl-OPFRs, TCPP and TDCPP, predominated among all of the measured OPFRs. Together with TCEP, ∑_3_Cl-OPFRs (4106 ± 1285 pg m^–3^) accounted for 82.7% of the total concentrations of 13 OPFRs (∑_13_OPFRs). The five alkyl-OPFRs, i.e., TPP, TEP, TnBP, TEHP, and TBOEP, accounted for another 10.7% (∑_5_Alkyl-OPFRs, 530 ± 234 pg m^–3^), and the rest were from the five aryl-OPFRs. Our results were similar to those observed in 10 Chinese cities,^27^ where Cl-OPFRs accounted for 77.0% of the total OPFRs measured. Multiple factors contribute to the concentration profile of OPFRs in PM_2.5_, including their physicochemical properties, emission sources, air-particle–water-soil distribution, atmospheric transport, and aging.^28^ Cl-OPFRs are the most widely used OPFRs globally, accounting for 24.0% of all OPFRs produced in 2020,^4^ and they usually appear in plastic films, furniture, electronics, automotive interiors, and rubber products. In addition, Cl-OPFRs are semivolatile (e.g., the vapor pressure of TCPP is 5.64 × 10^–5^ mmHg at 25 °C) and ready to partition onto PM_2.5_. With the chloro-substitutions, they are reluctant to atmospheric transformations through alkaline hydrolysis and photodegradation^29^ and relatively persistent in the environment (e.g., TCPP could persist for 1910 h in the environment).^10,28^ Therefore, PM_2.5_-bound Cl-OPFRs are able to migrate to remote areas through long-range transport.^28^

**Figure 1:**
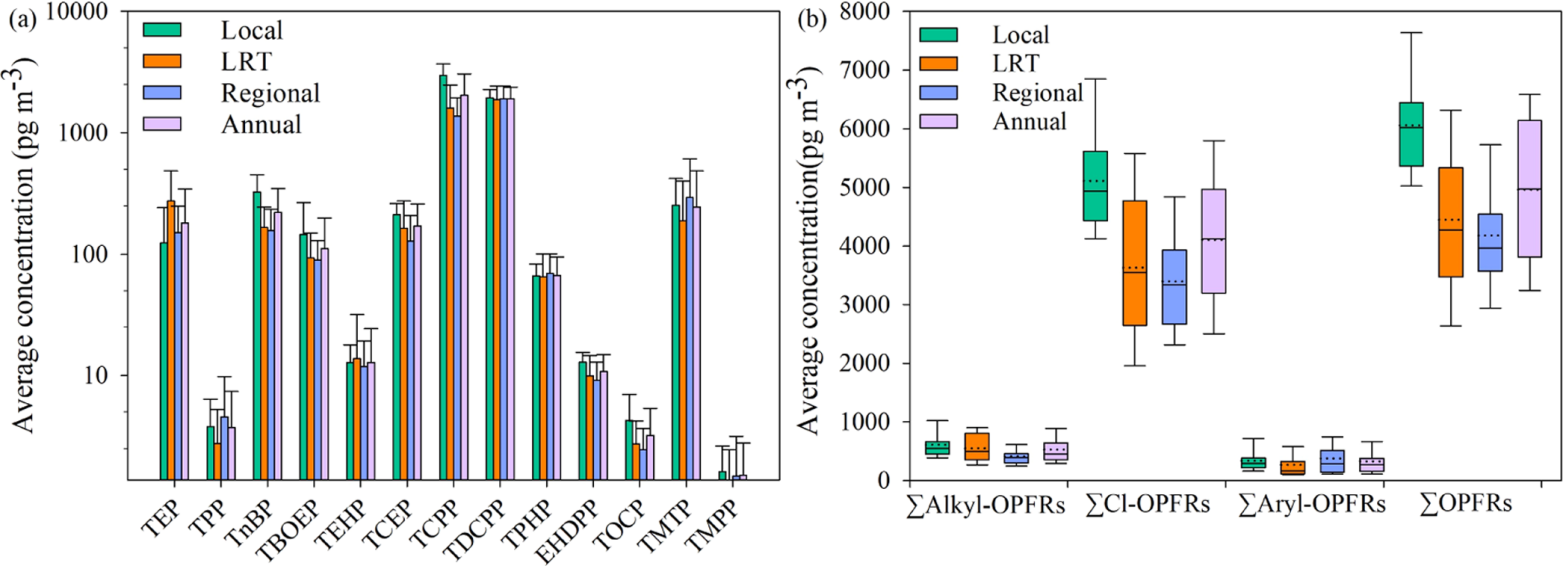
Average concentrations of individual OPFRs (a) and the summed concentrations of alkyl-OPFRs, Cl-OPFRs, aryl-OPFRs, and total OPFRs (b) in Hong Kong PM_2.5_ samples on local days, long-range transport (LRT) days, regional days, and during the whole year (annual). Boxplots in panel (b) show the descriptive statistics of the measured concentrations of PM_2.5_-OPFRs. Each subfigure shows the mean (dotted lines), median (solid lines), 25th and 75th percentile (boxes), and 5th and 95th percentile (whiskers).

Our previous studies have shown that the ambient levels of gas- and aerosol-phase pollutants in Hong Kong are greatly influenced by air mass origins.^30−33^ Average concentrations of the 13 PM_2.5_-bound OPFRs under local/marine pollution days, regional pollution days, and LRT pollution days are shown in Table S5 and Figure 1. Significant differences were observed among these three kinds of sampling days (*p* < 0.05), with the highest concentrations on local days (6062 ± 894 pg m^–3^, range: 4772–8481 pg m^–3^), followed by LRT days (4450 ± 1210 pg m^–3^, range: 2408–7664 pg m^–3^) and regional days (4181 ± 1165 pg m^–3^, range: 1924–7685 pg m^–3^). It is worth noting that higher OPFR loadings and ∑OPFRs/PM_2.5_ ratios were observed on local days when ambient PM_2.5_ levels were low (Figure S4), and both of them were significantly negatively correlated with PM_2.5_ mass (e.g., *R* = −0.783, *p* < 0.01 for ∑OPFRs/PM_2.5_). This suggests a clear local/marine source of OPFRs in Hong Kong since the air quality on local days is mainly influenced by local emissions and marine air. Temperature is another influential factor in the emission of OPFRs. Higher temperature not only accelerates the off-gassing of OPFRs from the OPFR-containing materials into the atmosphere but also aids the off-gassing of OPFRs from PM_2.5_. Higher levels of PM_2.5_-bound OPFRs were observed in summer than in winter in Great Lakes, USA,^34^ and Dalian, China.^35^ We also observed a clear seasonal variation with higher levels of OPFRs in the summer (5723 ± 923 pg m^–3^) than in the winter (4451 ± 1403 pg m^–3^) (Figure S5). Moreover, there is a positive correlation between OPFRs and temperature (*R* = 0.355, *p* < 0.01), indicating that the much higher amount of OPFRs in summer was from some local/marine emission sources, such as the solid waste treatment sites.

### 3.2 Source Apportionment of OPFRs

We applied the US Environmental Protection Agency (EPA) Positive Matrix Factorization (PMF) model 5.0 to apportion the sources of OPFRs. Details of the input species and the QA/QC analysis of PMF results are discussed in the SI (Text S4). The final results of source apportionment from the PMF analysis are shown in Figure S6. The first factor was identified as a secondary formation source based on the high fraction of monoterpenes SOA tracers. The second factor was dominated by the abundance of metals (i.e., Mn, Fe, Zn), representing the industrial emission source. Given that about 80% of levoglucosan was distinguished in the third factor, it was assigned as a biomass-burning source. The fourth factor was classified as vehicle emissions since hopanes were mainly resolved in this factor. The high loading of phthalates in the fifth factor made it a source of plastic processing and waste disposal.^36,37^ Almost 100% V and Ni were allocated in the sixth factor, regarded as marine vessels.^31^ It is worth noting that waste disposal is not waste incineration. Terephthalic acid was input into PMF in the preliminary model analysis, and it was resolved in the same factor as levoglucosan, in which a negligible amount of OPFRs was resolved. Moreover, a strong correlation was observed between terephthalic acid and levoglucosan (*R* = 0.727, *p* < 0.01), indicating that most of the terephthalic acid in the region may come from biomass burning. This is also in line with the fact that wastes in Hong Kong are landfilled rather than incinerated.^38^

### 3.3 Source-Specific Contributions to OPFRs under Different Meteorological Conditions

**Figure 2:**
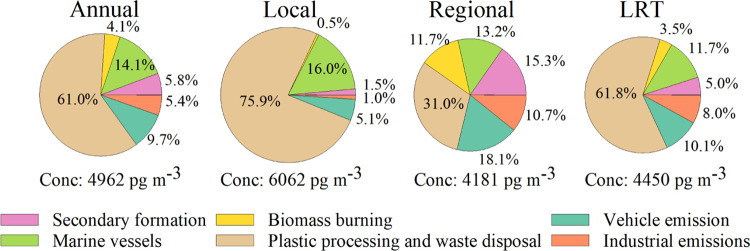
Source-specific contributions to OPFRs on local days, long-range transport (LRT) days, regional days, and during the whole year (annual) (mean concentrations are provided under the pie chart).

As shown in Figure 2, plastic processing and waste disposal source was the leading contributor to OPFRs throughout the year (61.0%, 3027 ± 1841 pg m^–3^), followed by marine vessels (14.1%, 698 ± 551 pg m^–3^), vehicle emissions (9.7%, 479 ± 553 pg m^–3^), secondary formation (5.8%, 289 ± 465 pg m^–3^), industrial emissions (5.4%, 266 ± 621 pg m^–3^), and biomass burning (4.1%, 202 ± 321 pg m^–3^).

Plastic processing and waste disposal accounted for 75.9% of the total OPFRs on local days (4604 ± 1081 pg m^–3^) (Figure 2). Although it also made the most significant contribution on regional days, the total emission dropped remarkably to 1297 ± 1085 pg m^–3^, accounting for 31.0% of all OPFRs. The stark differences in OPFRs source profiles between local and regional days indicated that plastics (e.g., plastic films and rubber) and waste (e.g., furniture waste including plastic cabinets for televisions and bedding, foam cushions, electronics like personal computers, and small appliances) bore primary responsibility for OPFR emissions in Hong Kong, especially on local days. As shown in Figure S6, most OPFRs were mainly apportioned to this source in PMF, especially for TCEP, TCPP, EHDPP, and TnBP. TCPP and TPHP are mainly applied in polyurethane foam;^39,40^ EHDPP is primarily used in food packaging, paints, and rubber,^8,40^ while TMPP and TBOEP are used in abandoned automobiles and electric wires.^41^ Like our results, Wang et al.^42^ also found waste recycling as an important source of environmental chlorinated- and aryl-OPFRs by measuring dust and soil samples collected from a waste recycling area. High concentrations of TCPP, TnBP, and TPHP were found in waste recycling areas in Pakistan^7^ and Qingyuan,^9^ illustrating the contribution of waste recycling to OPFRs. Since OPFRs are physically coated on the materials, they are prone to be released into the environment through volatilization and abrasion during the production and application processes.^43^ Moreover, 14 chlorinated-, alkyl-, and aryl-OPFRs were found in landfill leachate and sediment, indicating the leaching of OPFRs from landfilled waste.^44^ Since many electronic, electroplating, and plastic industries are located in the PRD region,^45^ a considerable amount of OPFRs may be transported to Hong Kong on regional and long-range transport days. However, in recent years, many unauthorized waste recycling facilities in the PRD region have been closed under stricter policy restrictions in mainland China.^46^ In contrast, to meet the increasing demand for a larger capacity of plastic processing and waste recycling in the region, the Hong Kong government has established several local waste recycling centers since 2005,^18^ such as the Kowloon Bay waste recycling center and EcoPark recycling center, which in turn lead to an increase of local emissions of OPFRs. In addition, most plastic waste is disposed of in the three strategic landfills in Hong Kong.^47^ Therefore, landfills are also potential contributors to ambient OPFRs.

Marine vessels were the second leading source of OPFRs in Hong Kong, especially on local days. As shown in Figure S6, considerable portions of TnBP, TBOEP, TOCP, TMTP, and TMPP were apportioned to this source in PMF. TMPP and TPHP are added to engine oils as plasticizers in marine vessels.^41^ TnBP, TBOEP, and TEHP are important components of hydraulic fluids, lubricants, and coatings, and they can off-gas and be abraded from marine machinery and equipment.^48^ Studies from Beibu Gulf in the South China Sea showed that emissions from fishery activities, especially fishing vessels, significantly contributed to OPFRs in coastal water.^16^ Gao et al.^49^ reported that the occurrence of OPFRs in water was affected by both local ship contamination and ocean transport. In our study, we first revealed that marine vessels are a vital source of PM_2.5_-bound OPFRs. On local days, the wind blowing over the ocean brought OPFRs emitted from marine vessels into Hong Kong. Therefore, 970 ± 547 pg m^–3^ of OPFRs were from marine vessels on local days, almost double those on regional days (553 ± 519 pg m^–3^) and LRT days (521 ± 445 pg m^–3^).

Vehicle emissions were the third leading contributor to OPFRs in Hong Kong during the entire year (Figure 2). However, unlike marine vessels and plastic processing and waste disposal sources, OPFRs from vehicle emissions were more of regional origin. It exhibited a higher contribution to OPFRs on regional days (756 ± 740 pg m^–3^, 18.1%) than on LRT days (448 ± 376 pg m^–3^, 10.1%) and local days (307 ± 426 pg m^–3^, 5.1%). Previous studies suggested TEP as an indicator of petrol-fueled vehicle emissions.^8^ Hu et al.^41^ summarized that high loads of TCPP, TPHP, and TBOEP originated from automotive interiors and rubber products (the main component of vehicle tires). Another study from New York also found that TCPP, TEP, and TBOEP exhibited high levels in air samples collected from automobile parts shops.^50^ Obviously, the increasing load of vehicles in the PRD region (e.g., there were more than 3 million vehicles in Shenzhen in 2016)^51^ leads to higher usage of rubber tires and automotive interiors. As a result, OPFRs emitted from vehicles and tire abrasions could be brought into Hong Kong by regional air mass, leading to higher contributions of vehicle emissions to PM_2.5_-bound OPFRs on regional days.

Although very limited studies have reported the secondary formation of atmospheric OPFRs, PMF results in our study indicated that 5.8% of PM_2.5_-bound OPFRs in Hong Kong were of secondary origin (Figure 2). The contribution of secondary formation to OPFRs was more significant on regional days (15.3%, 641 ± 678 pg m^–3^) than on LRT days (5.0%, 221 ± 193 pg m^–3^) and local days (1.5%, 90.0 ± 227 pg m^–3^). This observation is consistent with the higher levels of air oxidants (such as NO*_X_* and O_3_) observed on regional days (Table S2), which resulted in faster atmospheric oxidations and secondary formation from the potential volatile organic chemical precursors of OPFRs. For example, tris(2-chloroethyl) phosphite (TCEPi), tris(2-ethylhexyl) phosphite (TEHPi), 2-ethylhexyl diphenyl phosphite (EHDPPi), and triphenyl phosphite (TPHPi), which are organophosphite antioxidants (OPAs) used to retard the oxidation of polymers, may serve as the precursors of secondary formation of OPFRs in the atmosphere.^52^ The phosphorus atom in these OPAs could be oxidized by the atmospheric oxidants to generate the corresponding OPFRs (i.e., TCEP, TEHP, EHDPP, and TPHP).^52^ Moreover, some investigations have also reported that four novel OPFRs (i.e., tris(2,4-di*tert*-butyl phenyl) phosphate, bis(2,4-di*tert*-butyl phenyl) pentaerythritol diphosphate, tri-isodecyl phosphate, and tris(nonylphenyl) phosphate) were secondarily generated from the supplemental OPAs through the oxidation of phosphorus atoms during the production and processing of plastic polymers (e.g., the mulch film in farmlands).^19,53,54^ However, the mechanisms of the secondary formation of other OPFRs are not clear yet.

Industrial emissions have been found as a common source of OPFRs. It was reported that about 3228–4452 kg of atmospheric OPFRs were emitted annually from industrial-related sources in Guangzhou.^45^ TCPP, TMTP, and TEP are applied as cutting fluid and engine oil in various electronic equipment widely used in machine factories.^41^ Wang et al.^9^ investigated the concentration trends of PM_2.5_-bound OPFR from 20 sites (urban and rural) in Guangzhou. They found that it was consistent with the spatial distribution of industrial activities, indicating the key responsibility of manufacturing industries for OPFR emission in the PRD region. However, the industrial contribution to the OPFRs in Hong Kong was minor. It showed a clear regional origin with higher contributions on regional days (446 ± 919 pg m^–3^, 10.7%) and LRT days (354 ± 588 pg m^–3^, 8.0%) than on local days (60.0 ± 70.0 pg m^–3^, 1.0%), suggesting the regional transport of industrial-emitted OPFRs from the PRD region to Hong Kong. Biomass burning had the least contribution to OPFRs in Hong Kong, only 4.1% (202 ± 321 pg m^–3^). Similar to secondary formation sources, it was mainly from regional pollution (Figure 2).

### 3.4 Health Risk Assessment for PM_2.5_-Bound OPFRs

We followed the USEPA’s guidelines and evaluated the noncarcinogenic and carcinogenic risks of PM_2.5_-bound OPFRs to assess their exposure risk through human inhalation. The noncarcinogenic risks (NCR) of PM_2.5_-bound OPFRs were examined by calculating the estimated daily intake (EDI) and hazard quotient (HQ) of OPFRs.^41,55^
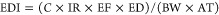
1
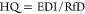
2where *C* is the ambient concentration of PM_2.5_-bound OPFRs (ng m^–3^), and two exposure scenarios, i.e., mean and 95th percentile concentrations, were examined to assess the average and high inhalation of OPFRs, respectively. IR is the inhalation rate (m^3^ day^–1^); EF is the exposure frequency (days/years); ED is the exposure duration (years); BW is the body weight (kg); and AT is the average time of exposure (days).

AT = ED × 365 days/year for the noncarcinogenic risk of human exposure.

AT = LT × 365 days/year for the carcinogenic risk of human exposure (assuming LT, lifetime, is 70 years).

RfD is the reference dose for OPFRs (ng kg-bw^–1^ day^–1^, Table S6). Considering that the OPFRs’ exposure for children and adults differs, we evaluated the health risks of PM_2.5_-bound OPFRs for children and adults separately. The IR, EF, ED, and BW values were obtained from Highlights of the Chinese Exposure Factors Handbook (2015)^56^ and are listed in Table S7, where a HQ value larger than 1 indicates a significant probability of noncarcinogenic risk upon OPFR exposure.^15^

The carcinogenic risk (CR) of PM_2.5_-bound OPFRs is calculated as follows
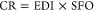
3where SFO is the oral cancer slope factor ((ng kg-bw^–1^ day^–1^)^−1^, Table S6). A CR value higher than 1 × 10^–6^ indicates a high probability of a person developing cancer from the lifetime exposure.^15^

**Figure 3:**
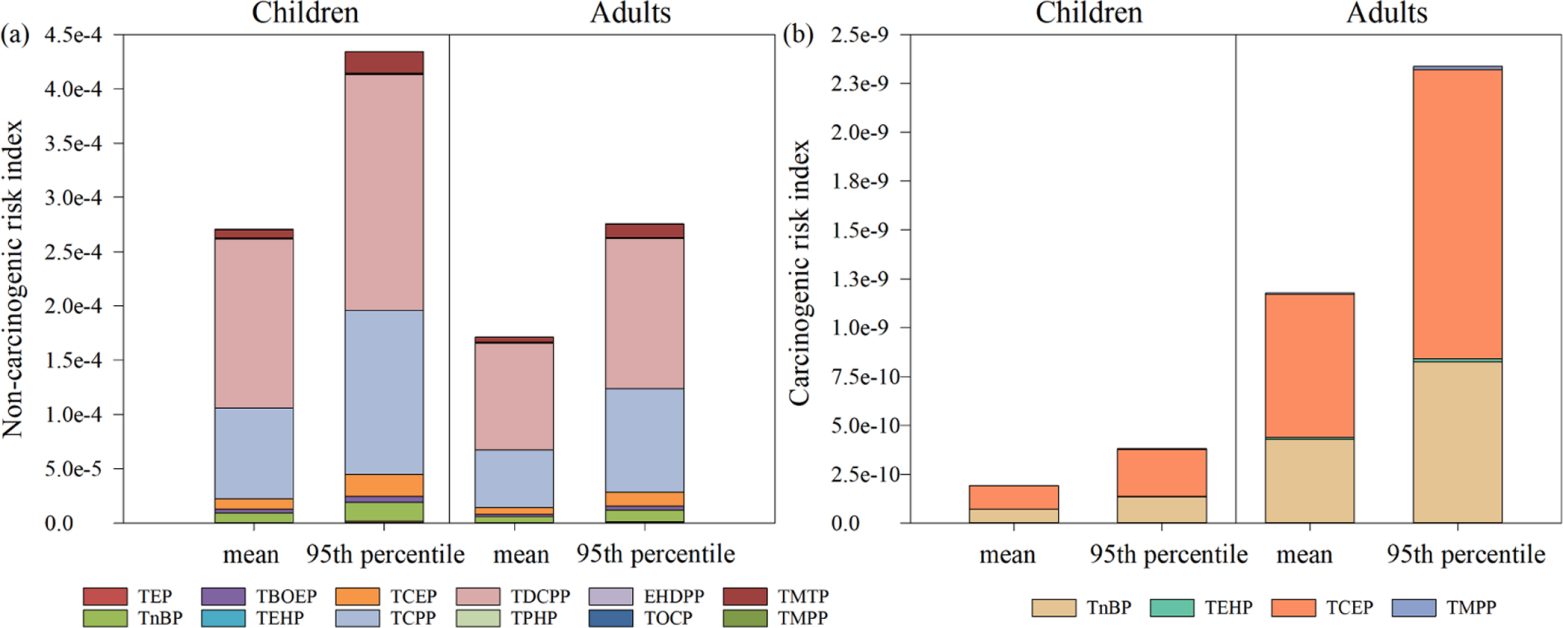
Noncarcinogenic risk index (a) and carcinogenic risk index (b) of PM_2.5_-bound OPFRs for children and adults via inhalation exposure under two scenarios.

The assessment results of NCR from OPFR exposure for children and adults are displayed in Figure 3a. The total NCR values based on the mean and 95th percentile level were 2.70 × 10^–4^ and 4.34 × 10^–4^ for children and 1.71 × 10^–4^ and 2.75 × 10^–4^ for adults, respectively, which are much lower than the threshold value of 1, indicating low exposure noncarcinogenic risk. Previous studies from inland Chinese cities, such as Guangzhou, Taiyuan,^8^ and Tianjin,^55^ also suggested minimum health risks posed by inhaling PM_2.5_-bound OPFRs. The NCR value for children was 1.5 times that for adults, attributed to children’s lighter body weights and higher exposure circumstances, and is worthy of attention. The risk rankings of OPFRs for children and adults are the same: TDCPP > TCPP > TCEP > TnBP > TMTP > TBOEP > TEP > TPHP > EHDPP > TOCP > TEHP > TMPP, of which Cl-OPFRs accounted for 92.1% of the NCR index for both children and adults under the mean exposure scenario, with 57.6% from TDCPP and 30.8% from TCPP. Previous studies have reported the noncarcinogenic risk (e.g., liver toxicity, reproductive toxicity, and neurotoxicity) of TCPP and TDCPP.^4,15,57^ Apart from their higher ambient levels than other OFPRs, the much higher NCR values of TCPP and TDCPP were also ascribed to their low RfD values, indicating their low acceptable risk levels. Thus, many countries and organizations (such as the U.S. and EU) have imposed restrictions on the application of TCPP and TDCPP in recent years, especially in some children’s products.^58,59^

The CR results of four OPFRs for children and adults are listed in Figure 3b. The total CR values for children and adults are below the threshold value (1 × 10^–6^) under both exposure scenarios. Similar to our results, Sun et al.^60^ also found that the carcinogenic risk of OPFRs in indoor dust in northern China cities was below the threshold. Different from noncarcinogenic risk, the CR values for adults are higher than those for children, probably due to the longer exposure duration (ED) in the whole lifetime for adults. The risk ranking of the four OPFRs is TCEP > TnBP > TEHP > TMPP. Studies have presented the potential carcinogenic risk of TCEP in the kidney^61^ and TnBP in the bladder.^62^ Apart from these four OPFRs, TCEP and TDCPP have also been reported to pose carcinogenic risks for humans and have even been restricted to use in the EU and part of the U.S. owing to their known carcinogenicity.^4^ However, the SFO data of TDCPP, TCPP, and other potential carcinogenic OPFRs are absent, leading to underestimation of the total CR index of human exposure to PM_2.5_-bound OPFRs calculated in this study.

### 3.5 Source-Specific Health Risk Assessment of PM_2.5_-Bound OPFRs

**Figure 4:**
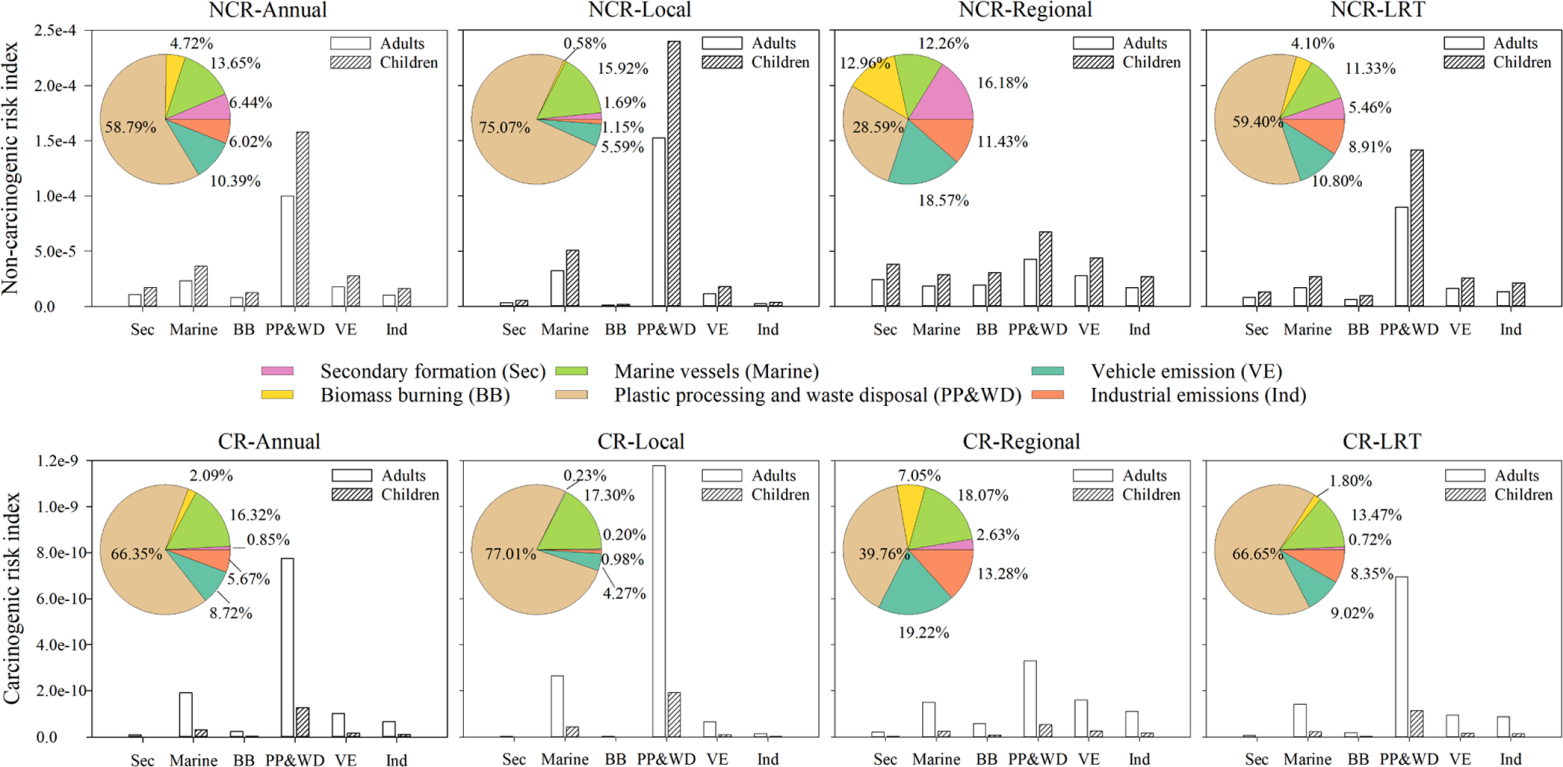
Source-specific contributions to the noncarcinogenic risk and carcinogenic risk of adults and children under different meteorological conditions and throughout the whole year.

To understand the source-specific health risks of human exposure to PM_2.5_-bound OPFRs, the average concentration of each OPFRs on annual/local/regional/LRT days apportioned to each source by PMF was applied to eqs 1–3 to calculate the associated NCR and CR of that OPFR in each source under different meteorological conditions. Then, the NCR and CR values of total OPFRs in each source were summed and are plotted in Figure 4 for adults and children separately. As shown in Figure 4, plastic processing and waste disposal (PP&WD, 58.8%), marine vessels (Marine, 13.7%), and vehicle emissions (VE, 10.4%) were the major sources contributing to the NCR of PM_2.5_-bound OPFRs for both children and adults annually, which is consistent with the source contribution profiles of OPFRs. More specifically, the NCR of all OPFRs for adults (under the mean exposure scenario, 2.00 × 10^–4^) and children (under the mean exposure scenario, 3.20 × 10^–4^) was the highest on local days, indicating a relatively higher health risk from local emissions. The two primary local emission sources of OPFRs, i.e., plastic processing and waste disposal as well as marine vessels, took the primary responsibility for NCR with a lumped contribution of 1.85 × 10^–4^ for adults and 2.91 × 10^–4^ for children on local days (both under mean exposure scenarios). The pronounced contribution of plastic processing and waste disposal to the health risks of OPFR emission should be brought to public attention. While on regional days, the combined contributions of vehicle emissions and industrial emissions to the NCR of all OPFRs were around three times as much as that on local days, indicating higher exposure risks of OPFRs emitted from these two sources in the PRD region. High loads of vehicles in the PRD region^51^ have raised significant concerns about the exposure risk of OPFRs from vehicle emissions (tires, braking, and automotive inferiors). Moreover, many large-scale industries and manufacturers (e.g., Japan’s biggest car parts maker, Denso) are located in the PRD region, resulting in significant health concerns for human exposure to OPFRs from industrial emissions.^63^

Similar to NCR, the main sources of CR were plastic processing and waste disposal (66.4%), marine vessels (16.3%), and vehicle emissions (8.7%) for both children and adults annually (Figure 4). Secondary formation (0.85%), biomass burning (2.1%), and industrial emissions (5.7%) comprised small contributions to the total CR. The CR value of total OPFRs was the highest on local days (1.50 × 10^–9^ for adults and 2.50 × 10^–10^ for children under mean exposure scenarios). The two local emission sources, plastic processing and waste disposal and marine vessels contributed a substantial proportion of 94.0% to the carcinogenic risks for both children and adults. This result can be explained by the high loadings of TCEP and TnBP allocated to the two main sources (plastic processing and waste disposal as well as marine vessels) and the potential carcinogenic risk of these two OPFRs.^61,62^ In addition, the proportion that plastic processing and waste disposal contributed to CR is consistently slightly higher than that to NCR under each meteorological condition, indicating a more threatening carcinogenic health risk of OPFRs from plastic processing and waste disposal.

### 3.6 Policy Implications

The relatively higher risk of PM_2.5_-bound OPFRs from plastic processing and waste disposal on local days is worthy of our attention. The significant difference between local and regional emission sources may be related to the different focus on waste management in Guangdong and Hong Kong. Since 2010, Guangdong province has proposed policies to regulate the environment of waste recycling and closed several waste cycling stations in the following years.^46^ While in Hong Kong, the number of waste recycling facilities has kept growing since 2005,^18^ leading to an expanding waste recycling capacity,^64^ which subsequently increases the emission of OPFRs on local days. Moreover, three key strategic landfills in the region receive thousands of tonnes of waste daily,^47^ contributing large amounts of OPFRs to the environment. Thus, emission regulations on plastic processing and waste management stations are critical to reducing the ambient level of OPFRs in Hong Kong and further minimizing their health risks.

Given the abundance and potential health risks of PM_2.5_-bound Cl-OPFRs in Hong Kong, it is necessary to restrict their production and applications. On the one hand, management policies can be implemented, including the collection of toxicological data on OPFR exposure (especially for vulnerable populations such as children), informing and drawing consumers’ attention, restrictions on the use of OPFRs with high concerns (e.g., TCEP and TDCPP), and so on. Many countries or organizations have regulated the applications of OPFRs. For example, the EU banned using Cl-OPFRs in electronic cases in 2018.^65^ Many states in the U.S., e.g., California^66^ and Maine,^67^ limited all OPFRs to 1000 ppm in children’s products, mattresses, and furniture in 2018. Canada prohibited using Cl-OPFRs in children’s loose-fitting sleepwear in 2016.^68^ On the other hand, manufacturers should look for innovative technologies to minimize the flammability of electronic enclosures, plastic material, and furniture, thereby reducing the addition of OPFRs in the products.

## Supporting Information Available

Chemical analysis methods (Text S1); optimization of the analytical method (Text S2); GC-APCI-MS/MS parameters and validation of the analytical method (Text S3); QA/QC of PMF analysis (Text S4); abbreviations, full names, formulas, and physicochemical parameters of OPFRs (Table S1); summary of meteorological information under local, long-range transport and regional influence (Table S2); optimized ion transitions for analysis of OPFRs by APGC-MS/MS with MRM mode (Table S3); calibration curves and LODs/LOQs of 13 OPFRs (Table S4); concentration range, mean value, and median value (pg m^–3^) of OPFRs in PM_2.5_ in Hong Kong (*n* = 65) (Table S5); exposure dose-response parameters (Table S6); parameters used in estimated daily intakes of PM_2.5_-bound OPFRs via inhalation (Table S7); corona current optimization for OPFRs (Figure S1); recoveries for OPFRs using different extraction solvents (a) and elution solvents (b) (Figure S2); recoveries for OPFRs at three different spiked levels (Figure S3); temporal variation of PM_2.5_ mass concentration, ∑_13_OPFR concentration, and normalized ∑_13_OPFR concentration (Figure S4); seasonal variations of total OPFRs, Cl-OPFRs, aryl-OPFRs, and alkyl-OPFRs (Figure S5); and distribution of OPFRs among six PMF-resolved sources (Figure S6) (PDF)
