# Supplementary material for: PM2.5-Bound Organophosphate Flame Retardants in Hong Kong: Occurrence, Origins, and Source-Specific Health Risks
Source: Environ Sci Technol. 2023 Sep 11;57(38):14289–98. doi: 10.1021/acs.est.3c04626 (PMC10537441; doi:10.1021/acs.est.3c04626)
Supplement: Supplementary file 1 — es3c04626_si_001.pdf [file es3c04626_si_001.pdf]

Supporting Information for

$PM_{2.5}$-Bound Organophosphate Flame Retardants in Hong Kong: Occurrence, Origins, and Source-Specific Health Risks

Xuemei Wang[1], Chin Wai Leung[1], Zongwei Cai[1,2], Di Hu[1,2,3]*

1. Department of Chemistry, Hong Kong Baptist University, Kowloon Tong, Kowloon, Hong Kong 999077, P. R. China
2. State Key Laboratory of Environmental and Biological Analysis, Hong Kong Baptist University, Kowloon Tong, Kowloon, Hong Kong 999077, P. R. China
3. HKBU Institute of Research and Continuing Education, Shenzhen Virtual University Park, Shenzhen 518057, P. R. China

* Email: dihu@hkbu.edu.hk. Tel: 852-34117859. Fax: 852-34117348.

Summary: 19 pages, 6 figures, 7 tables.

Contents:

Text S1 Chemical Analysis Methods

Text S2 Optimization of the Analytical Method

Text S3 GC-APCI-MS/MS Parameters and Validation of the Analytical Method

Text S4 QA/QC of the PMF Analysis

Table S1 Abbreviations, full names, formulas, and physicochemical parameters of OPFRs

Table S2 Summary of meteorological information under local, long-range transport and regional influence

Table S3 Optimized ion transitions for the analysis of OPFRs by APGC-MS/MS with MRM mode

Table S4 Calibration curves and LODs/LOQs of 13 OPFRs

Table S5 Concentration range, the mean value, and the median value (pg m^{-3}) of OPFRs in $PM_{2.5}$ in Hong Kong (n=65)

Table S6 Exposure dose-response parameters

Table S7 Parameters used in the estimated daily intakes of $PM_{2.5}$-bound OPFRs via inhalation

Figure S1 Corona current optimization for OPFRs

Figure S2 Recoveries for OPFRs using different extraction solvents (a) and elution solvents (b)

Figure S3 Recoveries for OPFRs at three different spiked levels

Figure S4 Temporal variations of $PM_{2.5}$ mass concentration, $\sum_{13}$OPFRs concentration, and normalized $\sum_{13}$OPFRs concentration

Figure S5 Seasonal variations of total OPFRs, Cl-OPFRs, aryl-OPFRs, and alkyl-OPFRs

Figure S6 Distribution of OPFRs among six PMF-resolved sources

Text S1 Chemical Analysis Methods

EC and OC were analyzed following the Improve A protocol using a DRI Model 2001 thermal/optical carbon analyzer (Sunset Laboratory, Oregon).[1] Five metal species (V, Ni, Mn, Fe, Zn) were analyzed by inductively coupled plasma mass spectrometry (ICP-MS, Agilent 7900, USA).[2] 18 hopanes (i.e., 22,29,30-trisnorneophopane, 22,29,30-trisnorphopane, αβ-norhopane, 22,29,30-norhopane, αα- + βα-norhopane, αβ-hopane, αα-hopane, βα-hopane, αβS-homohopane, αβR-homohopane, αβS-bishomohopane, αβR-bishomohopane, 22S-trishomohopane, 22R-trishomohopane, 22S-tretrahomohopane, 22R-tetrashomohopane, 22S-pentashomohopane, 22R-pentashomohopane) and five phthalates (diethyl phthalate, di-n-butyl phthalate, butyl benzyl phthalate, bis(2-ethylhexyl)phthalate, di-n-octyl phthalate) were quantified by thermal desorption GC-MS (Agilent 6890N-5975C, USA) analysis.[3] A GC-MS method with prior chemical derivatization was used to determine levoglucosan and biogenic SOA tracers.[4] Concentrations of major species in $PM_{2.5}$ and key gas pollutants were listed in Table S2.

Text S2 Optimization of the Analytical Method

Since the ionization efficiency of analytes is directly affected by the sample cone voltage and corona needle current in the APCI ionization source, these two mass spectrometry parameters were optimized to enhance the peak signal response and thus increase the sensitivity of OPFRs. We adjusted the values of cone voltage in the range of 5 V to 45 V and corona current from 1.5 μA to 5.5 μA for each OPFR compound (Figure S1). The optimized conditions are listed in Table S3. In the APCI ionization source, the molecular ion $[M]^{+\cdot}$ of each OPFR is the most dominant and was chosen as the precursor ion in the MS/MS analysis. The signal intensities of the product ions under different collision energies (5 V to 50 V) were monitored in the product ion scan mode. The top two most intensive product ions were selected as the characteristic quantitative and qualitative ions of each OPFR compound. The finalized MRM transitions and their collision energies for all OPFRs and internal standards in APGC-MS/MS analysis are listed in Table S3.

This study used ultrasonication to extract OPFRs from $PM_{2.5}$ samples, followed by purification on the Florisil column. To get higher recoveries of OPFRs in sample pretreatment processes, we tested a variety of solvents for sample extraction and elution. We tried four different solvents, i.e., DCM, DCM: hexane (1:1, v/v), DCM:

hexane (2:1, v/v), and acetone: hexane (1:1, v/v), to extract the blank quartz fiber filters spiked with 50 μL of mixed OPFRs standards (conc. in hexane). The extraction recoveries of OPFRs by these four solvents are shown in Figure S2a. For the purification step, four different elution solvents, i.e., ethyl acetate, acetone, DCM: acetone (1:1, v/v), and DCM: acetone (1:2, v/v), were tested to elute OPFRs from the Florisil column, and the results are shown in Figure S2b. We eventually selected DCM as the extraction solvent and DCM: acetone (1:1, v/v) as the elution solvent, given that the recoveries of almost all OPFRs under these conditions were 70%−120%, except for TEP. This proves that the sample pretreatment method of $PM_{2.5}$-bound OPFRs developed in this study is effective and efficient.

Text S3 GC-APCI-MS/MS Parameters and Validation of the Analytical Method

OPFRs were identified and quantified using an Agilent 7890B gas chromatograph (Agilent Technologies Inc.) equipped with a Xevo TQ-S triple quadrupole mass spectrometer (Waters, U.K.). The injection volume was 1 μL in the splitless mode. Temperatures of the injector, ion source, and transfer line were set to be 280°C, 230°C, and 300°C, respectively. Target contaminants were separated using a 30 m of HP-5MS column (0.25 mm i.d., 0.25 μm film thickness, Agilent, U.S.) at a constant flow rate of 1.5 mL min^{-1}. The GC oven temperature program was as follows: 70ºC for 1 min, 15ºC min^{-1} to 200ºC and held for 2 min, 15ºC min^{-1} to 300ºC, and remained for 6 min.

Calibration curves of all OPFRs were obtained, and most OPFRs detected by APGC-MS/MS showed good linearity between 0.02 ppb and 800 ppb with the correlation coefficient $R^2 > 0.99$ (Table S4). Besides, recovery and precision data were determined using blank filter samples spiked with mixed OPFRs standards at three levels (2 ppb, 10 ppb, and 100 ppb). The spiked samples were pretreated using the same procedures as for $PM_{2.5}$ samples, and each level was analyzed in triplicate. As a result, the recoveries of OPFRs were in the range of 70%−120% (Figure S3), with relative standard deviations (RSDs) less than 20%.

The instrumental LODs and LOQs for all OPFRs were calculated by 3 and 10 times the signal-to-noise ratio (S/N) of the quantitative ions in 7 injections of blank filters, which were 0.11−25.97 ppt and 0.35−82.58 ppt, respectively (Table S4). Method LODs and LOQs were obtained from the analysis of $PM_{2.5}$ samples through the sample pretreatment process, ranging from 0.03 to 0.65 pg m^{-3} and 0.10 to 2.06

pg m^{-3}, respectively. We achieved lower instrumental LODs (i.e., 0.11 ppt) for most OPFRs than those obtained by EI-MS (0.43 ppt).[5] Therefore, considering the trace concentrations of OPFRs in $PM_{2.5}$, the APGC-MS/MS-based analytical method developed in this study is appealing to quantitatively analyze OPFRs in $PM_{2.5}$ with high accuracy, sensitivity, selectivity, and robustness.

Text S4 QA/QC of the PMF Analysis

Twenty-five species were input into PMF, including 13 OPFR species, Σ_{13}OPFRs, EC, OC, and nine source markers (i.e., Ni, V, Mn, Fe, Zn, lumped hopanes, lumped phthalates, summed monoterpenes SOA tracers, and levoglucosan). The uncertainty values of the input species were set based on previous studies.[1,4] We tested 5−7 factors in the preliminary runs. Eventually, we determined a six-factor solution given that it satisfied all PMF QA/QC requirements and provided the best model interpretability and agreement between the observed and PMF-predicted concentrations for OPFRs (R^2 = 0.90). The Q_{Robust}/Q_{True} ratio of 100 base runs had a minimum Q value of 1.00. The scaled residuals for almost all species were normally distributed between −3 and 3, indicating no influence of outliers on the solution. To ensure the stability of the solution, displacement (DISP) assessment and 100 bootstraps run with a minimum correlation R-value of 0.6 were performed. The DISP summary showed no observed drop of Q, and there was no error and no swaps for dQ_{max}=4 either, suggesting a stable and reliable solution.

Table S1. Abbreviations, full names, formulas, and physicochemical parameters of OPFRs

Compounds	Abbreviation	CAS#	M.W.	Formula	Solubility at 25℃ (mg L^{-1})	Vapor pressure at 25℃ (mmHg)	Log K_{OW}	log K_{OA}	Structure
Triethyl phosphate	TEP	78-40-0	182.15	$C_6H_{15}O_4P$	5E5	0.29	0.8	6.63	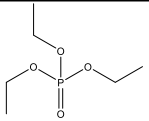
Tripropyl phosphate	TPP	513-08-6	224.23	$C_9H_{21}O_4P$	6E3	0.029	1.87	6.43	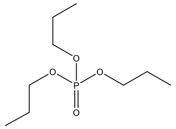
Tri-n-butyl phosphate	TnBP	126-73-8	266.31	$C_{12}H_{27}O_4P$	280	0.0011	4	8.24	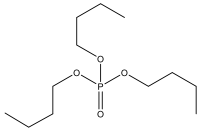
Tris(2-chloroethyl) phosphate	TCEP	115-96-8	285.49	$C_6H_{12}Cl_3O_4P$	7000	1.6E−5	1.44	5.31	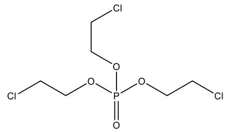
Tris(1-chloro-2-propyl) phosphate	TCPP	13674-84-5	327.56	$C_9H_{18}Cl_3O_4P$	1200	5.64E−5	2.59	8.2	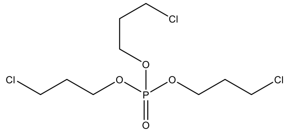
Tris(1,3-dichloro-2-pro pyl) phosphate	TDCPP	13674-87-8	430.9	$C_9H_{15}Cl_6O_4P$	7	2.61E−9	3.27	10.6	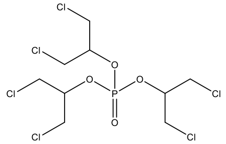
Tris(2-butoxyethyl) phosphate	TBOEP	78-51-3	398.5	$C_{18}H_{39}O_7P$	1100	1.2E−6	3.75	13.1	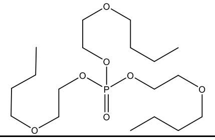

Triphenyl phosphate	TPHP	115-86-6	326.3	$C_{18}H_{15}O_4P$	1.9	4.7E−7	4.59	8.46	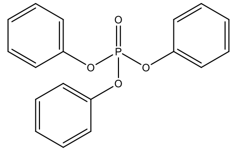
2-Ethylhexyl diphenyl phosphate	EHDPP	1241-94-7	362.4	$C_{20}H_{27}O_4P$	0.067	6.5E−7	5.73	8.38	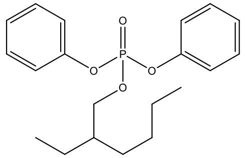
Tris(2-ethylhexyl) phosphate	TEHP	78-42-2	434.6	$C_{24}H_{51}O_4P$	0.6	8.25E−8	8.95	15	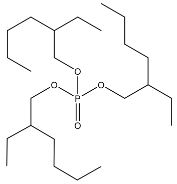
Tri-o-cresyl phosphate	TOCP	78-30-8	368.4	$C_{21}H_{21}O_4P$	0.3	1.1E−7	5.11	12	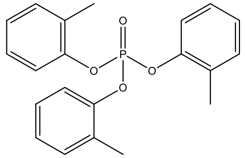
Tri-m-tolyl phosphate	TMTP	563-04-2	368.4	$C_{21}H_{21}O_4P$		7.8E−7	5.11	12	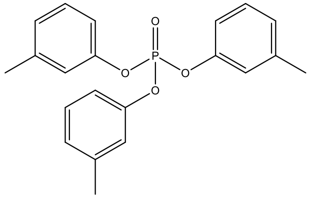
Tri-p-cresyl phosphate	TMPP	78-32-0	368.4	$C_{21}H_{21}O_4P$	0.074	4.9E−7	5.11	12	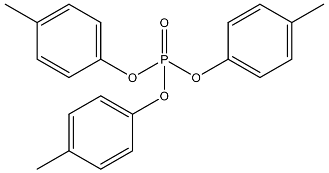

M.W.=Molecular Weight; K_{OW}=Octanol-Water Partition Coefficient; K_{OA}=Octanol-Air Partition Coefficient.

Table S2. Summary of meteorological information under local, long-range transport, and regional influence

	Local Days			Long Range Transport Days			Regional Days			Annual		
	Mean	Median	Range	Mean	Median	Range	Mean	Median	Range	Mean	Median	Range
Temperature (℃)	27.9	28.5	22.2-30.5	21.5	20.5	11.5-30.5	21.5	20.2	15.2-32.7	23.8	24.6	11.5-32.7
Relative Humidity (%)	82.6	80.5	73.2-96.6	79.1	80.4	59.7-91.2	70.6	73.0	53.0-89.5	77.8	78.9	53.0-96.6
O_3 (ppb)	10.12	8.47	1.25-36.41	22.50	20.94	4.92-50.70	28.93	27.62	5.24-60.41	19.93	14.49	1.25-60.41
NO_2 (ppb)	24.03	21.13	14.19-46.51	28.54	28.98	16.70-54.21	32.91	32.47	23.06-46.26	28.21	27.94	14.19-54.21
SO_2 (ppb)	3.10	2.65	1.60-6.38	2.39	1.95	0.95-7.60	3.36	3.17	1.10-5.37	2.93	2.60	0.95-7.60
$PM_{2.5}$ (μg m^{-3})	17.45	14.06	6.54-49.48	25.28	26.42	10.59-45.89	37.73	34.57	12.55-70.60	25.86	26.42	6.54-70.60
OC/EC and Specific Markers for Source Apportionment												
OC (μg m^{-3})	2.13	1.90	1.11-6.45	3.75	3.20	1.34-9.80	5.69	5.44	2.29-11.51	3.69	3.12	1.11-11.51
EC (μg m^{-3})	1.28	1.11	0.61-2.58	1.32	1.39	0.65-2.82	2.29	1.98	0.76-4.23	1.60	1.47	0.61-4.23
V (ng m^{-3})	10.44	9.92	0.85-23.92	4.95	3.43	0.36-18.69	5.20	3.74	0.87-17.20	7.03	5.75	0.36-23.92
Ni (ng m^{-3})	5.11	4.67	1.25-12.11	2.98	2.29	0.18-10.00	3.39	2.12	0.92-10.88	3.85	3.12	0.18-12.11
Mn (ng m^{-3})	2.54	1.77	0.52-9.06	8.51	5.27	1.10-36.14	12.54	7.76	0.77-48.72	7.61	4.45	0.52-48.72
Fe (ng m^{-3})	55.10	49.14	7.67-146.01	105.75	99.32	20.95-305.94	144.62	128.48	33.62-396.47	99.60	85.32	7.67-396.47
Zn (ng m^{-3})	34.58	24.45	7.69-87.70	135.18	62.00	11.36-806.83	234.68	58.61	7.96-1497.72	131.58	50.29	7.69-1497.72
Hopanes (ng m^{-3})	1.24	0.73	0.33-5.57	1.39	1.03	0.34-3.85	3.02	2.46	0.68-7.53	1.84	1.02	0.33-7.53
Phthalates (ng m^{-3})	100.77	91.49	16.02-189.33	108.29	97.34	18.40-406.15	130.47	100.76	23.33-414.61	112.34	98.11	16.02-414.61
Monoterpene (ng m^{-3})	10.56	3.48	1.69-105.16	21.24	21.51	2.26-48.04	57.19	46.48	8.36-159.63	28.47	18.91	1.69-159.63
Levoglucosan (ng m^{-3})	3.27	1.30	0.95-15.23	13.86	11.21	1.05-65.50	44.31	30.35	1.10-106.88	19.40	9.41	0.95-106.88

Table S3. Optimized ion transitions for the analysis of OPFRs by APGC-MS/MS with MRM mode

Compounds	Abbreviation	Retention time/min	Cone Voltage/V	Quantitative ions	CE/V	Qualitative ions	CE/V
Triethyl phosphate	TEP	5.45	35	182>99	20	182>155	5
Tripropyl phosphate	TPP	7.88	25	224>99	25	224>141	5
Tri-n-butyl phosphate-d_{27}	TnBP-d_{27}	9.90	15	293>102	20	293>229	10
Tri-n-butyl phosphate	TnBP	10.03	15	266>99	20	266>153	10
Tris(2-chloroethyl) phosphate-d_{12}	TCEP-d_{12}	11.04	35	298>131	15	297>261	10
Tris(2-chloroethyl) phosphate	TCEP	11.15	35	285>99	20	285>125	15
Tris(1-chloro-2-propyl) phosphate	TCPP	11.59	25	328>99	20	328>252	10
Tris(1,3-dichloro-2-propyl) phosphate	TDCPP	16.23	30	431>99	35	431>209	20
Tris(2-butoxyethyl) phosphate	TBOEP	16.63	15	399>99	40	399>225	10
Triphenyl phosphate-d_{15}	TPHP-d_{15}	16.62	40	341>243	15	341>223	25
Triphenyl phosphate	TPHP	16.67	40	326>215	30	326>325	5
2-Ethylhexyl diphenyl phosphate	EHDPP	16.81	5	362>251	20	362>250	10
Tris(2-ethylhexyl) phosphate	TEHP	16.98	30	435>99	50	435>81	50
Tri-o-cresyl phosphate	TOCP	18.08	30	368>165	30	368>91	40
Tri-m-tolyl phosphate	TMTP	18.23	5	368>165	40	368>243	30
Tri-p-cresyl phosphate	TMPP	18.56	40	368>165	35	368>197	35

Table S4. Calibration curves and LODs/LOQs of 13 OPFRs

Compounds	Calibration curve	Instrumental LOD pg mL^{-1}	Instrumental LOQ pg mL^{-1}	Method LOD pg m^{-3}	Method LOQ pg m^{-3}
TEP	y=0.0190x-1.558	1.96	6.25	0.64	2.02
TPP	y=0.001939x-0.02130	4.13	13.13	0.13	0.41
TnBP	y=0.00220x-0.2919	6.47	20.59	0.65	2.06
TCEP	y=0.4243x-52.79	0.74	2.36	0.09	0.27
TCPP	y=0.1032x+92.73	18.96	60.31	0.19	0.60
TDCPP	y=0.9441x+215.4	25.97	82.58	0.03	0.10
TBOEP	y=0.01908x-2.665	3.83	12.19	0.60	1.91
TPHP	y=0.6478x-8.607	0.83	2.64	0.26	0.82
EHDPP	y=0.04878x-3.540	0.51	1.62	0.32	1.02
TEHP	y=0.05779x-12.95	1.58	5.02	0.13	0.42
TOCP	y=0.5457x-15.19	0.11	0.35	0.23	0.74
TMTP	y=0.01154x-3.697	1.08	3.44	0.48	1.52
TMPP	y=0.4272x-25.67	0.14	0.45	0.10	0.33

Table S5. Concentration range, mean value, and median value (pg m^{-3}) of OPFRs in $PM_{2.5}$ in Hong Kong (n=65)

Compounds	Local Days		Long-Regional Transport Days		Regional Days		Whole Year	
(pg m^{-3})	Mean ± SD	Median (Range)	Mean ± SD	Median (Range)	Mean ± SD	Median (Range)	Mean ± SD	Median (Range)
TEP	124.44±116.69	91.71 (26.04-599.77)	274.42±210.09	210.40 (34.18-667.35)	149.57±98.46	131.08 (32.07-449.42)	180.63±163.02	112.39 (26.04-667.35)
TPP	3.79±2.58	3.15 (0.46-9.61)	2.76±2.47	2.05 (N.D.-11.11)	4.55±5.21	3.13 (N.D.-25.3)	3.73±3.66	2.89 (0.42-25.3)
TnBP	325.27±125.75	319.12 (162.43-682.48)	165.36±78.30	143.60 (31.29-411.5)	156.23±77.61	128.33 (65.31-381.47)	221.59±126.40	186.09 (31.29-682.48)
TBOEP	145.59±120.06	113.67 (73.98-701.5)	93.04±56.11	77.44 (40.24-234.23)	89.50±39.89	66.60 (49.82-177.84)	111.35±86.70	100.35 (40.24-701.5)
TEHP	12.74±5.17	13.90 (2.88-21.1)	13.73±18.06	6.61 (1.73-68.14)	11.81±7.34	10.19 (2.82-36.48)	12.77±11.51	9.97 (1.73-68.14)
∑Alkyl-OPFRs	611.82±239.44	547.42 (295.77-1335.49)	548.92±237.62	495.82 (214.62-1036.35)	411.44±164.04	387.63 (237.59-983.35)	529.84±233.67	452.78 (214.62-1335.49)
TCEP	211.50±48.97	212.39 (130.27-321.17)	163.75±110.47	129.21 (51.85-475.04)	128.06±79.32	109.33 (37.02-413.41)	170.40±89.18	153.62 (37.02-475.04)
TCPP	2963.33±719.13	2935.07 (1492.04-4707.99)	1603.56±867.33	1456.58 (367.79-3909.03)	1371.19±560.18	1332.15 (644.45-2642.33)	2034.13±1021.99	1903.64 (367.79-4707.99)
TDCPP	1938.38±338.84	1844.76 (1619.09-3083.5)	1864.80±550.01	1804.27 (922.24-3179.47)	1894.89±520.15	1953.55 (730.51-3083.88)	1901.23±473.63	1834.79 (730.51-3179.47)
∑Cl-OPFRs	5113.21±858.33	4938.33 (4009.3-6952.87)	3632.12±1215.70	3548.28 (1724.1-5849.78)	3394.14±987.09	3336.81 (1423.78-5946.39)	4105.76±1285.44	4118.63 (1423.78-6952.87)
TPHP	65.97±16.85	61.46 (42.50-103.49)	64.65±35.66	59.32 (28.28-174.54)	69.16±31.17	56.68 (26.27-139.27)	66.52±28.60	59.59 (26.27-174.54)
EHDPP	12.83±2.64	12.74 (6.48-18.62)	9.94±4.62	8.18 (4.82-20.68)	9.12±3.76	8.00 (3.81-19.18)	10.76±4.06	10.78 (3.81-20.68)
TOCP	4.26±2.73	3.04 (1.55-10.44)	2.73±1.49	2.57 (1.2-6.83)	2.45±1.20	2.01 (1.1-5.36)	3.21±2.14	2.58 (1.1-10.44)

TMTP	252.27±167.42	208.85 (52.91-658.44)	188.74±212.12	87.56 (18.36-951.93)	292.99±316.72	196.84 (48.43-1487.57)	244.27±239.79	175.06 (18.36-1487.57)
TMPP	1.61±1.01	1.29 (0.46-4.01)	1.37±1.07	1.04 (N.D.-4.82)	1.48±1.68	1.10 (N.D.-8.28)	1.50±1.27	1.13 (0.33-8.28)
∑Aryl-OPFRs	336.94±170.97	289.67 (112.95-731.28)	267.30±226.86	168.75 (88.45-1041.14)	375.13±331.19	287.08 (110.14-1584.09)	326.19±251.14	269.23 (88.45-1584.09)
∑OPFRs	6061.97±894.05	6025.17 (4772.38-8480.75)	4449.69±1210.22	4273.16 (2407.78-7664.19)	4180.71±1164.63	3969.25 (1924.09-7684.74)	4961.79±1380.39	4978.22 (1924.09-8480.75)
∑OPFRs/$PM_{2.5}$ (pg μg^{-1})	459.21±213.35	488.99 (96.45-877.81)	202.98±101.96	186.24 (78.89-491.64)	123.48±57.92	120.44 (40.62-304)	273.12±206.07	190.64 (40.62-877.81)

N.D. not detected

Table S6. Exposure dose-response parameters

Compounds	RfD ($\times 10^3$ (ng kg-bw^{-1} day^{-1})	SFO[d] ((ng kg-bw^{-1} day^{-1})$^{-1}$)
TEP	130[a]	-
TPP	-[c]	-
TnBP	10[a]	9×10^{-9}
TBOEP	15[b]	-
TEHP	100[a]	3.2×10^{-9}
TCEP	7[a]	2×10^{-8}
TCPP	10[a]	-
TDCPP	5[f]	-
TPHP	70[a]	-
EHDPP	15[e]	-
TOCP	13[b]	-
TMTP	13[b]	-
TMPP	13[b]	2×10^{-8}

a: Data were adopted from the United States Environmental Protection Agency (USEPA).[6]

b: Data were adopted from Van den Eede *et al*.[7]

c: There is no reference data.

d: Data were adopted from Azizi *et al.*[8]

e: Data were adopted from Poma *et al.*[9]

f: Data were adopted from the National Research Council.[10]

Table S7. Parameters used in the estimated daily intakes of $PM_{2.5}$-bound OPFRs via inhalation

Parameters	Children (6-12 years)	Adults
body weight (BW, kg)	22	62
exposure duration (ED, years)	6	58
Inhalation rate (IR, m^3 day^{-1})	9	16.1
exposure frequency (EF, days $year^{-1}$)	365	365
Average time exposure for carcinogenic risk (AT, days)	25550	25550
Average time exposure for non-carcinogenic risk (AT, days)	2190	21170

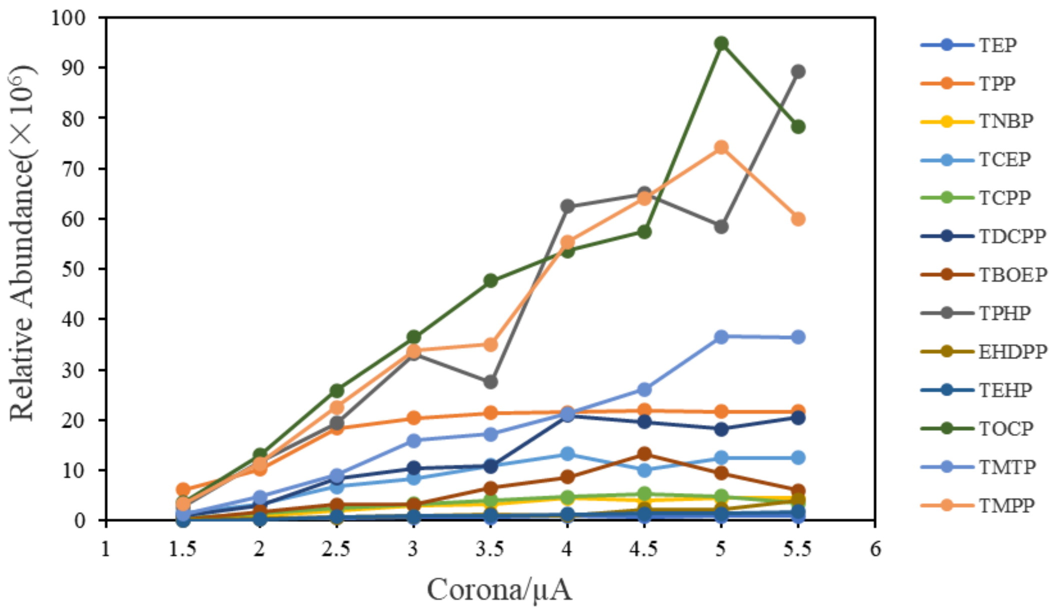

Figure S1. Corona current optimization for OPFRs.

(a)

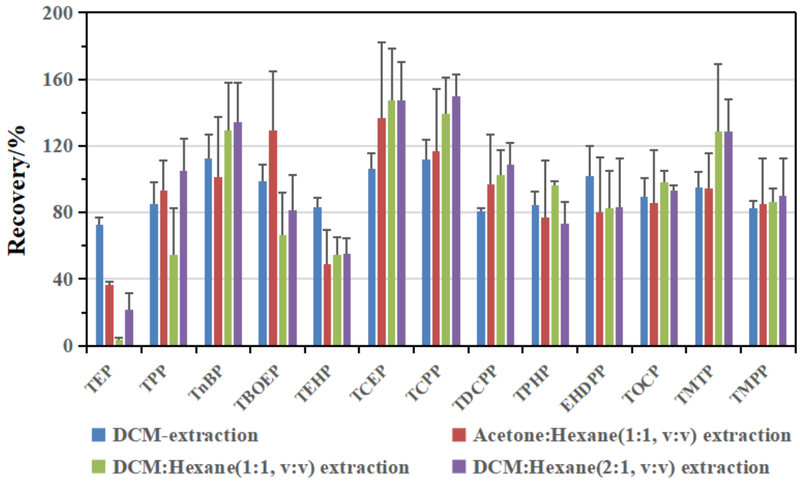

(b)

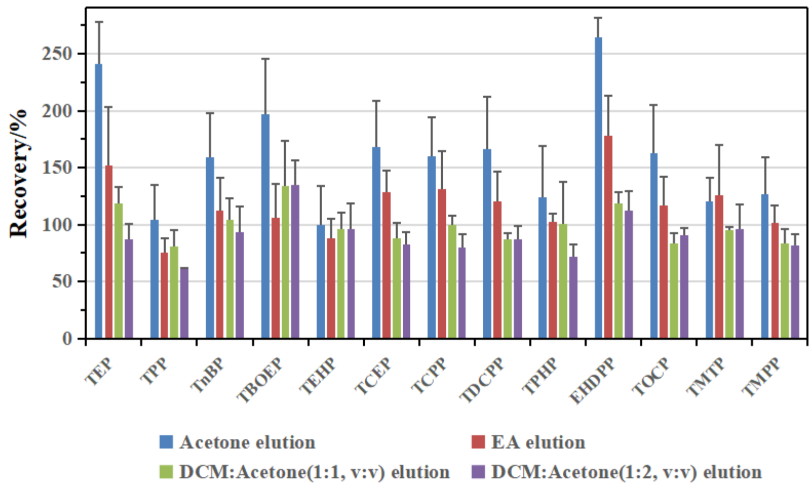

Figure S2. Recoveries for OPFRs using different extraction solvents (a) and elution solvents (b).

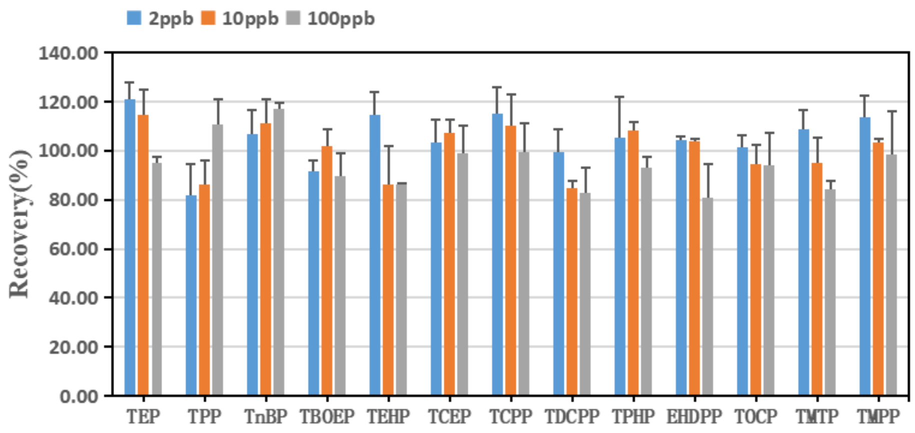

Figure S3. Recoveries for OPFRs at three different spiked levels.

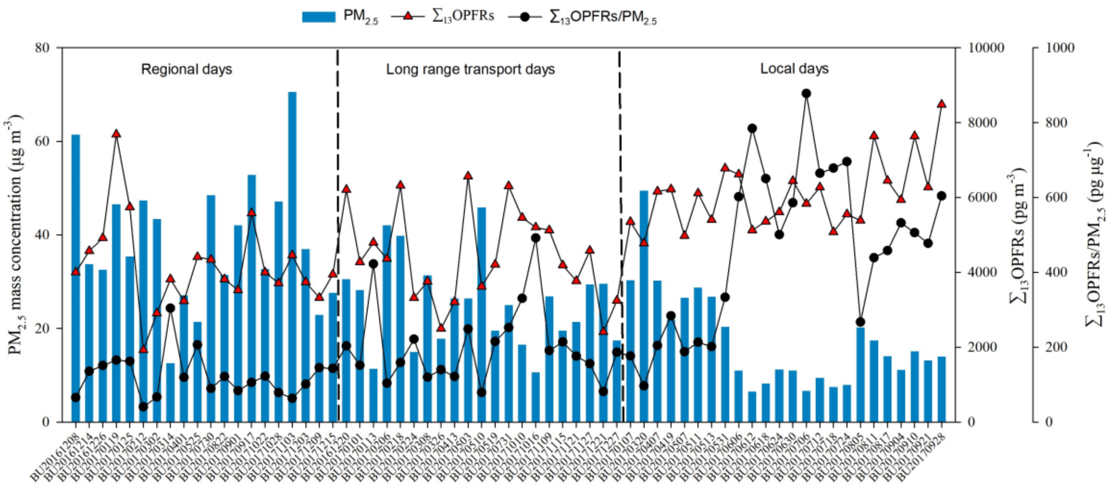

Figure S4. Temporal variations of $PM_{2.5}$ mass concentration, $\sum_{13}$OPFRs concentration, and normalized $\sum_{13}$OPFRs concentration.

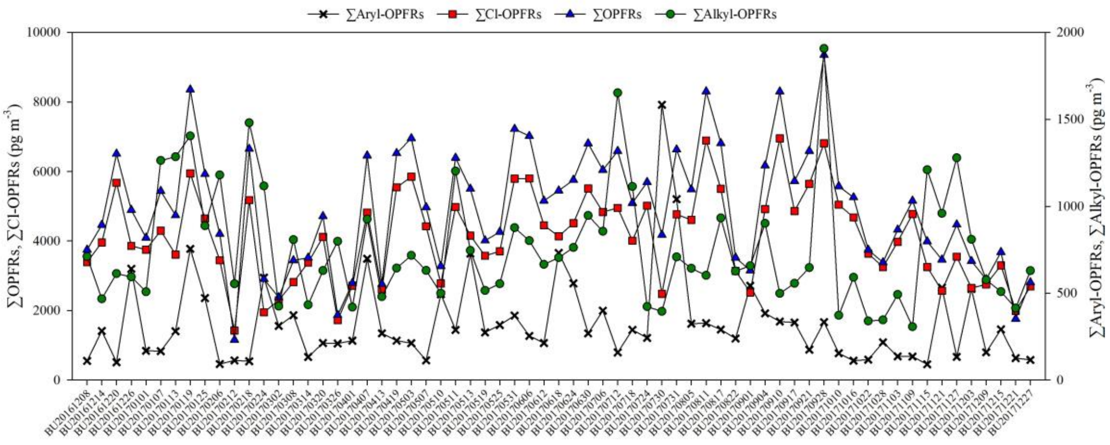

Figure S5. Seasonal variations of total OPFRs, Cl-OPFRs, aryl-OPFRs, and alkyl-OPFRs.

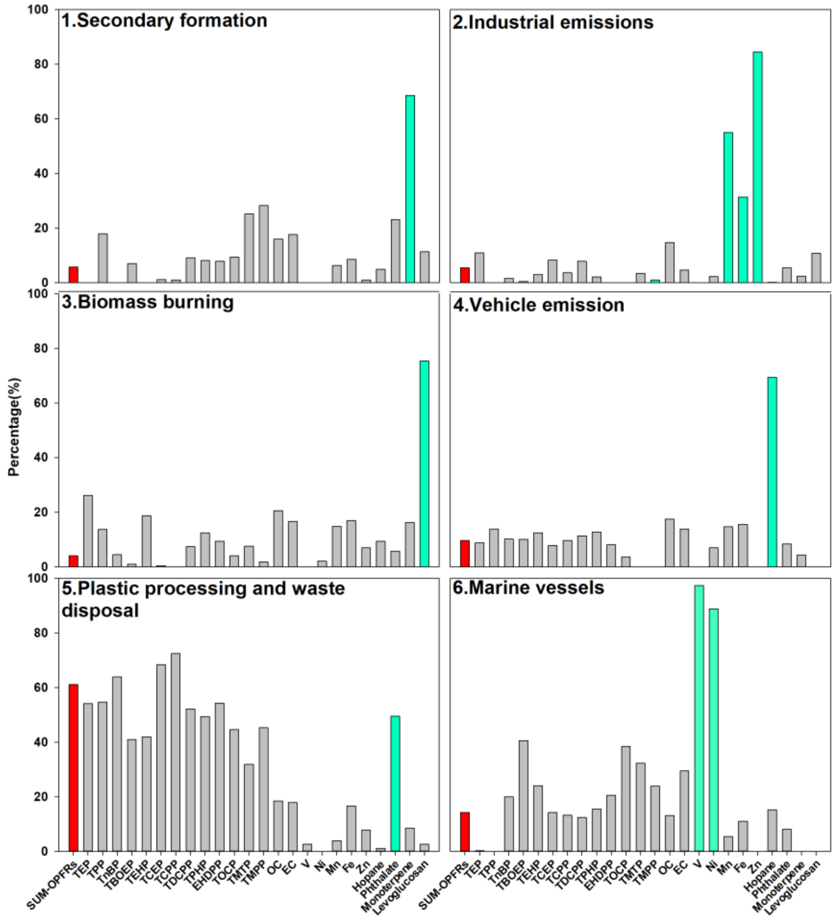

Figure S6. Distribution of OPFRs among six PMF-resolved sources.

References

(1) Hu, D.; Bian, Q.; Lau, A. K.; Yu, J. Source apportioning of primary and secondary organic carbon in summer $PM_{2.5}$ in Hong Kong using positive matrix factorization of secondary and primary organic tracer data. *J. Geophys. Res.* **2010**, *115,* D16204.

(2) Ma, Y.; Cheng, Y.; Qiu, X.; Cao, G.; Kuang, B.; Yu, J. Z.; Hu, D. Optical properties, source apportionment and redox activity of humic-like substances (HULIS) in airborne fine particulates in Hong Kong. *Environ. Pollut.* **2019**, *255*, 113087.

(3) Ho, S. S. H.; Yu, J. Z. In-injection port thermal desorption and subsequent gas chromatography-mass spectrometric analysis of polycyclic aromatic hydrocarbons and n-alkanes in atmospheric aerosol samples. *J. Chromatogr. A.* **2004**, *1059*, 121-129.

(4) Cheng, Y.; Ma, Y.; Dong, B.; Qiu, X.; Hu, D. Pollutants from primary sources dominate the oxidative potential of water-soluble $PM_{2.5}$ in Hong Kong in terms of dithiothreitol (DTT) consumption and hydroxyl radical production. *J. Hazard. Mater.* **2020**, 124218.

(5) Chen, Y.; Chen, Y. J.; Zhang, Y.; Li, R.; Chen, W.; Yan, S. C.; Qi, Z.; Chen, Z. F.; Cai, Z. Determination of HFRs and OPFRs in $PM_{2.5}$ by ultrasonic-assisted extraction combined with multi-segment column purification and GC-MS/MS. *Talanta* **2018**, *194*, 320-328.

(6) United States Environmental Protection Agency (USEPA), 2013. Mid Atlantic Risk Assessment, Regional Screening Levels (RSLs) - Generic Tables. http://www.epa. gov/region9/superfund/prg (accessed May, 2023).

(7) Van den Eede, N.; Dirtu, A. C.; Neels, H.; Covaci, A. Analytical developments and preliminary assessment of human exposure to organophosphate flame retardants from indoor dust. *Environ. Int.* **2011**, *37*, 454-61.

(8) Azizi, S.; Dehghani, M. H.; Naddafi, K.; Nabizadeh, R.; Yunesian, M. Occurrence of organophosphorus esters in outdoor air fine particulate matter and comprehensive assessment of human exposure: A global systematic review. *Environ. Pollut.* **2022**, 120895.

(9) Poma, G.; Sales, C.; Bruyland, B.; Christia, C.; Goscinny, S.; Van Loco, J.; Covaci, A. Occurrence of organophosphorus flame retardants and plasticizers (pfrs) in belgian foodstuffs and estimation of the dietary exposure of the adult population. *Environ. Sci. Technol*. **2018**, *52*, 2331-2338.

(10) National Research Council. Toxicological risks of selected flame-retardant chemicals. 2000.
